# The Treatment of Uterine Flexions

**Published:** 1886-12

**Authors:** 


### THE TREATMENT OF UTERINE FLEXIONS.

The *Medical Press and Circular*, of London, England, of November 3d, in an editorial review of Dr. Virgil O. Hardon's article on The Treatment of Uterine Flexions, which appeared in the August number of THE JOURNAL, credits the author with having explained certain phenomena in connection with flexions of the womb which have been noted by various observers, particularly Matthew Duncan, but of which no satisfactory explanation has heretofore been given. In the course of the review Dr. Hardon is alluded to as "evidently an original thinker," a compliment to which we think the article in question has justly entitled him. The English reviewer disapproves, however, of the treatment by the supporting tampon, on the ground that it necessitates too frequent vaginal interference. But if, as claimed by Dr. Hardon, the beneficial results of this mode of treatment are proportionate to the frequency of its application, we fail to see the force of the objection urged against it.

